# ProFace: a server for the analysis of the physicochemical features of protein-protein interfaces

**DOI:** 10.1186/1472-6807-6-11

**Published:** 2006-06-07

**Authors:** Rudra P Saha, Ranjit P Bahadur, Arumay Pal, Saptarshi Mandal, Pinak Chakrabarti

**Affiliations:** 1 Department of Biochemistry, Bose Institute, P-1/12 CIT Scheme VIIM, Calcutta 700 054, India

## Abstract

**Background:**

Molecular recognition is all pervasive in biology. Protein molecules are involved in enzyme regulation, immune response, signal transduction, oligomer assembly, etc. Delineation of physical and chemical features of the interface formed by protein-protein association would allow us to better understand protein interaction networks on one hand, and to design molecules that can engage a given interface and thereby control protein function on the other hand.

**Results:**

ProFace is a suite of programs that uses a file, containing atomic coordinates of a multi-chain molecule, as input and analyzes the interface between any two or more subunits. The interface residues are shown segregated into spatial patches (if such a clustering is possible based on an input threshold distance) and/or core and rim regions. A number of physicochemical parameters defining the interface is tabulated. Among the different output files, one contains the list of interacting residues across the interface. Results can be used to infer if a particular interface belongs to a homodimeric molecule.

**Conclusion:**

A web-server, ProFace (available at ) has been developed for dissecting protein-protein interfaces and deriving various physicochemical parameters.

## Background

Most proteins function by interacting with other molecules; the binding sites have evolved for achieving specific interactions and avoiding undesirable associations that would be deleterious to the normal functioning of the cell. Thus the interfaces between two protein subunits provide context for understanding the principles of molecular recognition. A large volume of structural data on protein interactions, either complexes between independent polypeptide chains, or oligomeric assembly of subunits, is available in the Protein Data Bank (PDB) [1], which has been used to generate diverse datasets of protein-protein interfaces [2]. The physical and chemical features of the interfaces have been analyzed [3-8] and softwares/websites, such as Protein-Protein Interaction Server [6], MolSurfer [9], SPIN-PP [10], etc. are available for their calculations. Nevertheless, our understanding of the biomolecular interactions is not adequate enough, for example, to infer unambiguously the arrangement of the subunits in an oligomeric protein from crystallographic studies [11], or to ascertain a high success rate for the prediction of models of protein-protein complexes through docking methods [12].

Recently, protein-protein interfaces have been dissected from new perspectives [13,14]. It has been shown that many large interfaces are not contiguous, but built of spatially demarcated surface patches. Such segregation into patches is also indicative of the location and distribution of water molecules held in the interface [15]. Additionally, one can also divide the interface into core and rim regions using the difference of solvent accessibilities of residues and the chemical properties of each region are quite distinct. Interestingly, this division also mirrors the degree of conservation of interface residues in a family of homologous proteins [16], and this represents an important signature of protein interaction sites. Various other physicochemical parameters have also been developed [17,18], which in combination, can distinguish the true oligomeric state (dimer, in particular) from the lattice contacts observed in protein crystals. In this article we describe a web-server, ProFace that dissects a given protein-protein interface and obtains various parameters to characterize it.

## Implementation and results

### Input file and parameters

All the protein chains should be contained in the input file in the PDB format and the user must indicate which chains (a maximum of three allowed) constitute each of the two components forming the interface between them. Also, one has to specify the way to display the dissected interface, i.e., to show the residues belonging to core and rim and/or in spatial patches. For clustering into patches the threshold distance has to be supplied. This distance should typically be half the maximum distance between any two interface atoms on a given protein chain – the latter distance is listed along with the other parameters in the output. Ideally, the number of patches should be the same on both the components and if this is not the case the threshold value may have to be slightly changed (increase to reduce the number of patches and vice-versa) to achieve this. The suggested values are 15 Å for protein-protein complexes [13] and 22 Å for homodimers [14], as these gave patches that were visually meaningful in the vast majority of the cases.

### Output files and parameters

There are five types of output: a) plot of interface residues with secondary structural information; b) statistics of interface parameters; c) coordinates of interface atoms and the PDB files in which the interface residues are tagged; d) list of residue contacts across interface; and e) the view of the interface atoms.

#### Plot of interface residues with secondary structural information

**Figure 1:**
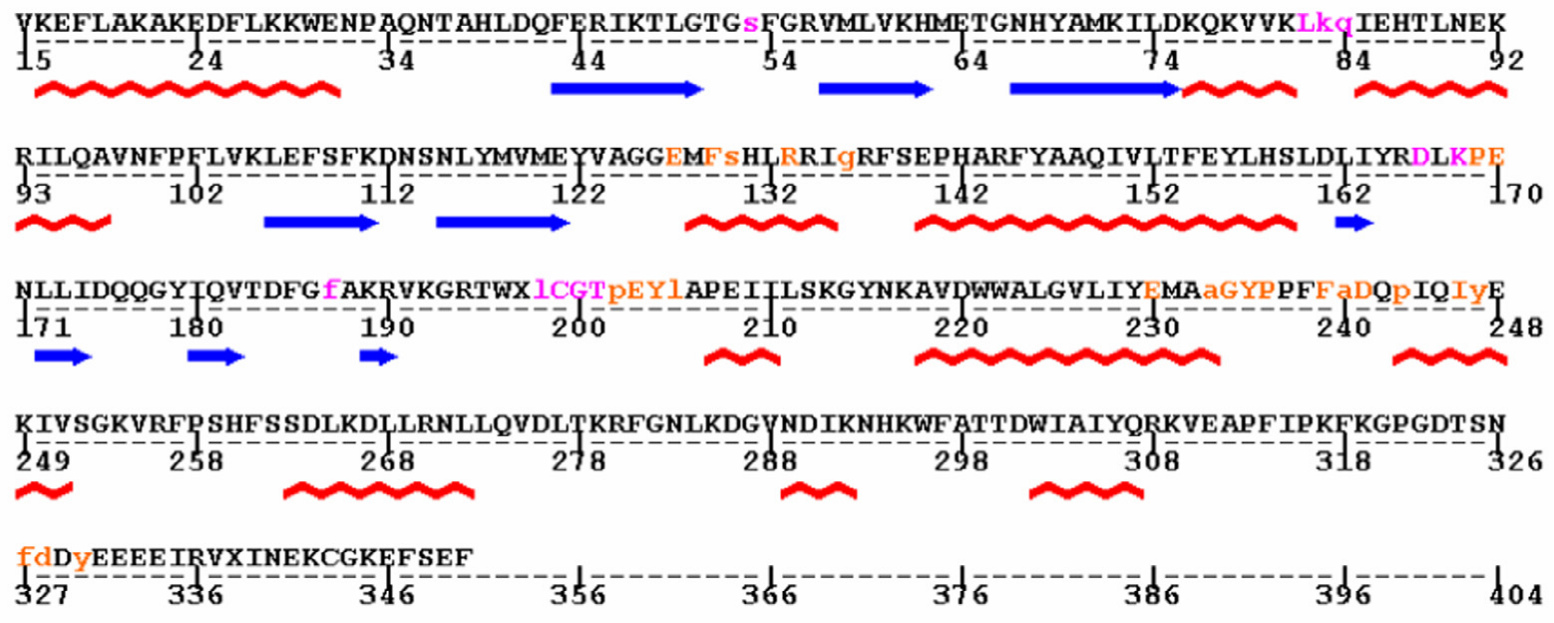
The interface residues shown against the sequence of c-AMP dependent protein kinase in complex with H7 protein kinase inhibitor 1-(5-isoquinolinesulfonyl)-2-methylpiperazine (PDB file, 1ydr) [24]. There are two patches and the residues belonging to them are shown in orange and magenta (in decreasing patch size). Core and rim residues are distinguished by upper and lower-case letters, respectively. An α-helix is represented by red undulation and a β-strand by blue arrow.

The secondary structural elements (α-helix and β-strand) are computed using the program DSSP [19] and shown below the residue names (one-letter code) along the sequence for the individual chains. The sequence information is based on residues for which coordinates are available (and not on the basis of SEQRES records). There are three options to show the interface residues: (i) to simply show the interface residues (in red color); (ii) to show them dissected into core/rim regions (red/blue color); and to show them dissected in two different ways – spatial patches (in different colors) and core/rim regions (upper/lower case). An example of option (iii) is displayed in Figure 1.

#### Statistics of interface parameters

A typical example of output parameters is shown in Table 1. The interface area is the sum of the solvent accessible surface areas (ASA) of the two components less that of the pair. ASA is calculated using program NACCESS [20]. All protein atoms or residues contributing more than 0.1 Å^2 ^to the interface area are counted as interface atoms or residues, whose numbers are tabulated. Non-polar interface area is the area contributed by non-polar interface atoms (i.e., all atoms excluding O, N and S). Interface area/surface area is the ratio of the interface area to the rest area of the protein surface in the two components. Fraction of non-polar atoms is based not on the area contributed, but on the number of atoms. Fraction of fully buried atoms is the ratio of interface atoms that are completely buried in the complex (with ASA = 0) to the total number of interface atoms (which also include atoms that do not have zero ASA in the complex). Residue propensity score and local density are defined in Bahadur et al. [17]. Residues with at least one fully buried interface atom are designated as core residues, while rim residues do not contain any interface atom that is fully-buried. Once a residue is identified as core, all its constituent atoms are assumed to be in core (irrespective of the atom being fully or partially buried) and the interface area contributed by the atoms of the residue is part of the core region. Statistics also include atoms/residues/areas divided into core and rim regions (Table 2). Also the number of patches in individual chains and their respective sizes are tabulated (Table 3).

#### Output files

The 4-digit code used to name the output files are randomly generated and does not have any correspondence to the input file name. The coordinates are stored in two types of files (with extensions .pdb and .int) and there are two files (corresponding to individual components) of each type. In the .pdb file the interface residues are distinguished from the remaining atoms in the structure on the basis of the content in the two columns – occupancy factor and B-factor. The non-interface residues have a value of 0.00 in these columns. For the interface residues, a) the occupancy is replaced by -5.00 (if it is a core residue) or 5.00 (if it is a rim residue); b) the B-factor column is replaced by a value 1.00 through 9.00, depending on the patch to which the residue belongs. In the .int file, only the interface atoms are kept, with the occupancy and the B-factor column modified as above (and an additional information on patches is also provided by appending labels a, b, c, ... to the keyword ATOM to correspond to patch numbers 1, 2, 3,...). Moreover, there are two additional columns, in which the ASAs of the constituent atoms in the individual component and in the complex are provided. One can use this information to calculate the interface area contributed by individual residues and, for example, correlate with the thermodynamic data on the free energy of binding [16].

**Figure 2:**
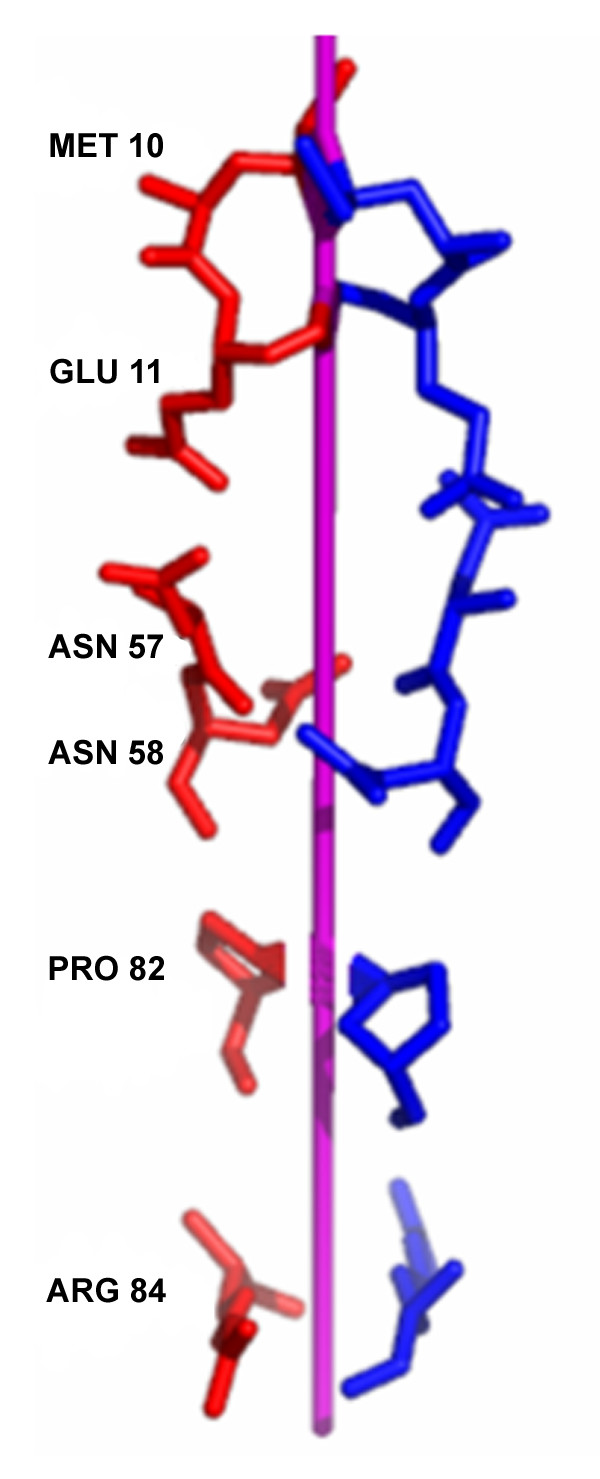
Self-contacting residues in the dimeric structure of wheat germ agglutinin (9wga) [25]. Residues in the two subunits are in two different colors (and those of one chain labeled), with the 2-fold axis running vertically.

Another output file (with extension .cont) provides the list of residue contacts across the interface. For an interface residue in the first component the list shows the interface residues from the other component which are within a distance of 4.5 Å. If a pair of residues in contact have the same residue name and number, this is indicated by the symbol '<< ---' at the end of the line. This interaction has been designated as self-contact and indicates that the interface may have been formed by components/chains related by a 2-fold symmetry [18]. An example of the presence of self-contacting residues in a homodimer structure is presented in Figure 2. Some of the parameters in Table 1, along with the information on self-contacting residues may be used to ascertain if a 2-fold related contact observed in a crystal structure truly represents a biological homodimer.

#### View of the interface atoms

This can be done using either RasMol [21] or CHIME [22], depending on whichever program has been configured by the user on the machine. Clicking on the RasMol link will first enable the user to download the PDB file (with interface atoms), which can then be viewed by either program. Clicking on the CHIME link loads the PDB file directly in CHIME. As the B-factor column of the PDB file has been replaced by number codes indicating the patch to which the atoms belong, the interface atoms can be colored on the basis of patches using RasMol. Also, the PDB file generated by the program can be used in GRASP [23] to color the molecular surface according to the criterion of patch or core/rim region.

## Conclusion

ProFace can be used to dissect a protein-protein interface, deriving physicochemical parameters. The output can be used to display the interface with standard softwares and understand the biological significance of the interaction.

## Availability and requirements

• Project name: ProFace

• Project home page: 

• Operating system(s): Platform independent

• Programming language: Java, C++

• Other requirements: JRE 1.4.2.04 or higher, Chime plug-in 2.6 or higher; all of them are available for download at the above web address

• License: Free

• Any restrictions to use by non-academics: None

## Authors' contributions

RPS and RPB wrote the source codes, participated in developing the server. AP and SM participated in developing the server. PC conceived the study, and participated in its design, analysis, and coordination. RPS, RPB, AP, SM and PC all contributed to writing the final manuscript and interpretation of data.

## Figures and Tables

**Table 1:** Interface parameters of c-AMP-dependent protein kinase complex (PDB code, 1ydr) [24]

	Component 1	Component 2	Total
Interface Area (Å^2^)	921.15	1076.27	1997.42
Interface Area/Surface Area	0.06	0.42	0.11
Number of atoms	115	87	202
Number of residues	36	18	54
Fraction of non-polar atoms	0.68	0.62	0.65
Non-polar interface area (Å^2^)	525.35	653.11	1178.46
Fraction of fully buried atoms	0.32	0.30	0.31
Residue Propensity Score	0.64	0.35	0.99
Local Density	39.57	40.51	

**Table 2:** Statistics on the core and rim regions of the interface in the file, 1ydr

Chain	Core			Rim			Total		
	
	Atoms	Residues	Area	Atoms	Residues	Area	Atoms	Residues	Area
E	37	20	623.80	78	16	297.35	115	36	921.15
I	26	9	884.52	61	9	191.75	87	18	1076.27

**Table 3:** Areas of individual patches in the interface of the two components in 1ydr

Chain	No. of patches	No. of residues in patches	Patch area (Å^2^)
E	2^a^	25,11	660.08, 261.07
I	2	13,5	725.78, 350.49

^a ^A threshold value of 16 Å was used to get two patches; the default value of 15 Å gave three.
